# Compendium of *Bifidobacterium*-based probiotics: characteristics and therapeutic impact on human diseases

**DOI:** 10.20517/mrr.2024.52

**Published:** 2024-10-08

**Authors:** Fabiana Bocchio, Leonardo Mancabelli, Christian Milani, Gabriele Andrea Lugli, Chiara Tarracchini, Giulia Longhi, Flora De Conto, Francesca Turroni, Marco Ventura

**Affiliations:** ^1^Department of Medicine and Surgery, University of Parma, Parma 43124, Italy.; ^2^Interdepartmental Research Centre “Microbiome Research Hub”, University of Parma, Parma 43124, Italy.; ^3^Laboratory of Probiogenomics, Department of Chemistry, Life Sciences and Environmental Sustainability, University of Parma, Parma 43124, Italy.; ^#^Authors contributed equally.

**Keywords:** Microbiota, microbiome, probiotic, beneficial bacteria, bifidobacteria, *Bifidobacterium*

## Abstract

The human microbiota, a complex community of microorganisms residing in and on the human body, plays a crucial role in maintaining health and preventing disease. *Bifidobacterium* species have shown remarkable therapeutic potential across a range of health conditions, thus being considered optimal probiotic bacteria. This review provides insights into the concept of probiotics and explores the impact of bifidobacteria on human health, focusing on the gastrointestinal, respiratory, skeletal, muscular, and nervous systems. It also integrates information on the available genetic bases underlying the beneficial effects of each bifidobacterial probiotic species on different aspects of human physiology. Notably, *Bifidobacterium*-based probiotics have proven effective in managing gastrointestinal conditions such as constipation, antibiotic-associated diarrhea, irritable bowel syndrome (IBS), inflammatory bowel disease (IBD), and *Helicobacter pylori* infections. These benefits are achieved by modulating the intestinal microbiota, boosting immune responses, and strengthening the gut barrier. Moreover, *Bifidobacterium* species have been reported to reduce respiratory infections and asthma severity. Additionally, these probiotic bacteria offer benefits for skeletal and muscular health, as evidenced by *Bifidobacterium adolescentis* and *Bifidobacterium breve*, which have shown anti-inflammatory effects and symptom relief in arthritis models, suggesting potential in treating conditions like rheumatoid arthritis. Furthermore, probiotic therapies based on bifidobacterial species have shown promising effects in alleviating anxiety and depression, reducing stress, and enhancing cognitive function. Overall, this review integrates the extensive scientific literature now available that supports the health-promoting applications of probiotic *Bifidobacterium* species and underscores the need for further research to confirm their clinical efficacy across different body systems.

## INTRODUCTION

Multicellular organisms, including humans, inhabit in association with a vast number of microorganisms, including bacteria, archaea, viruses, and unicellular eukaryotes. This complex microbial community, collectively known as the microbiota, is involved in a symbiotic relationship with its host. Within the human body, the microbiota exerts critical roles across a variety of physiological processes, including nutrient metabolism and immune system modulation^[1,2]^. These interactions are supposed to be vital for maintaining health and preventing disease, demonstrating the key role exploited by the microbiota in host well-being^[3-5]^.

The human body harbors distinctive microbial communities across its various anatomical sites, which are adapted to the unique environmental conditions of the specific body site^[6]^. Distinct microbiotas are found, for example, on the skin or internal body sites such as the mouth, the respiratory tract, the gastrointestinal tract, and the female reproductive tract, each fulfilling specialized functions^[7,8]^.

Due to its complexity and diversity, the intestinal microbiota represents a target of marked scientific interest among these diverse microbial habitats. This dense microbial community is considered to play a critical role in the host’s well-being, performing several beneficial functions. The intestinal microbiota is supposed to be involved in crucial metabolic processes, including carbohydrate digestion and vitamin synthesis^[1]^. Furthermore, it is supposed to play an important role in the development of immune responses and in safeguarding the host against colonization by pathogenic bacteria^[9,10]^.

The composition of the intestinal microbiota is influenced by a series of endogenous features closely related to the host and environmental factors, such as age, sex, diet, antibiotic treatment, and lifestyle^[11-14]^.

Maintaining the balance of the intestinal microbiota is crucial for health, as this symbiotic relationship can be disrupted by events that change the composition of the microbiota, leading to dysbiosis. Dysbiosis is characterized by an imbalance in the gut's microbial ecosystem^[15]^. Numerous studies have highlighted a correlation between intestinal dysbiosis and several human diseases/disorders, such as inflammatory bowel disease (IBD), irritable bowel syndrome (IBS), type 2 diabetes (T2D), celiac disease, and obesity^[10,16,17]^. In this context, preserving and restoring the intestinal microbiota balance is crucial for maintaining overall human health^[18]^.

**Figure 1:**
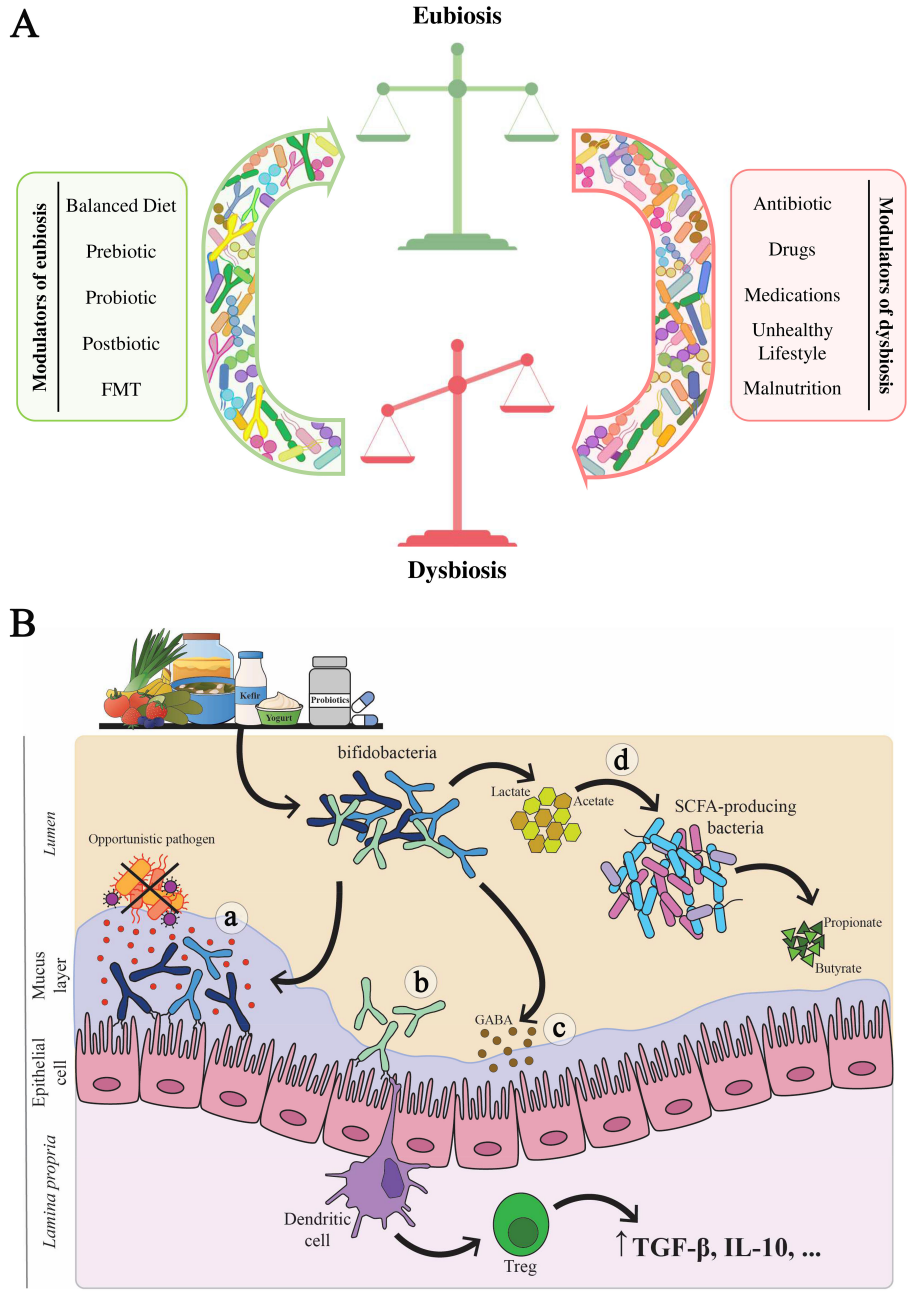
Illustration of potential modulators and general mechanisms influencing gut microbiota composition and host health. Panel (A) illustrates the potential modulators of the human microbiota. Specifically, modulators that promote eubiosis are highlighted in green, while modulators that contribute to dysbiosis are highlighted in red; Panel (B) shows several mechanisms through which bifidobacteria exert beneficial effects on the host. Specifically, bifidobacteria strengthen the intestinal barrier by increasing mucus production and preventing the colonization of opportunistic pathogens, thereby protecting against infections (a). Additionally, bifidobacteria interact with the host’s immune system, stimulating the production of anti-inflammatory cytokines that regulate immune responses and reduce inflammation (b). Bifidobacteria also produce neuroactive molecules, such as GABA, which may influence the nervous system, alleviating stress and supporting mental well-being (c). Furthermore, bifidobacteria generate metabolites like lactate and acetate, which can be utilized by other gut bacteria, such as SCFA-producing bacteria, to synthesize beneficial compounds like butyrate and propionate, contributing to gut health and metabolic regulation (d). GABA: Gamma-aminobutyric acid; SCFA: short-chain fatty acid.

Currently, this modulation can occur through various methods and interventions, each capable of influencing the composition and activity of the microbes within our bodies [Figure 1A]. Among these, diet plays an important role in modulating the intestinal microbiota. In fact, changes in dietary habits can profoundly impact the types of microbes residing in the gut. Thus, certain foods can promote the growth of beneficial bacteria, while others may promote the proliferation of harmful microorganisms^[19]^. In addition, probiotics and prebiotics, i.e., compounds that stimulate the growth of beneficial bacteria, are considered to play a crucial role in shaping the microbiota composition and promoting gut health^[20,21]^.

Moreover, in recent years, an innovative approach known as fecal microbiota transplantation (FMT) has emerged as a promising intervention for restoring healthy microbiota in individuals with gut disorders or conditions related to microbial imbalance^[22-28]^. In detail, FMT involves introducing processed fecal material from a healthy donor to the recipient to reintroduce a diverse array of beneficial microbes, thereby promoting gut health and overall well-being. Numerous studies have highlighted the efficacy of this treatment and its therapeutic potential across various pathologies, with the main target being the treatment for recurrent *Clostridioides difficile* infection^[29]^. Nonetheless, concerns about disease transmission, recipient immune response, potential adverse effects, lack of standardization, absence of unified legislation, high costs, and invasiveness emphasize the importance of further refining FMT as a therapeutic approach, making it still largely experimental in most cases^[30]^.

Among the various approaches modulating the intestinal microbiota, probiotic microorganisms are the most utilized due to their applicability and less invasive nature while still being effective. Probiotics aim to restore intestinal homeostasis by promoting the colonization of beneficial bacteria and restricting the expansion of pathogenic bacteria, thereby preventing dysbiosis conditions^[31]^. Lactobacilli and bifidobacteria are the most prevalent and widely used among all probiotic organisms^[32,33]^. Bifidobacteria are well-documented for their health benefits and have co-evolved with humans, playing a fundamental role in the early gut microbiota^[34,35]^. In fact, bifidobacteria are considered essential for “educating” the immune system stimulating and supporting gut physiology, such as mucin production^[36-38]^. Likewise, these microorganisms could inhabit the human gut due to the host’s production of prebiotic molecules like mucin, which supports their survival and colonization^[37,39]^. This relationship and the demonstrated transmission of these microorganisms from mother to child through vertical transmission mechanisms^[40]^ underscore the co-evolution of bifidobacteria with their human host. The decrease in this bacterial population within both children’s and adult’s gut microbiota has been negatively correlated with numerous diseases, including autoimmune diseases, IBD, and potentially cancer^[41-44]^. Thus, these microorganisms are considered crucial for maintaining gut health and overall homeostasis throughout adulthood^[45]^. The beneficial effects of bifidobacteria on human gut health and their role in maintaining a balanced microbiota make them a primary focus in probiotic research. Their ability to modulate the gut microbiota, improve intestinal barrier function, and exert anti-inflammatory effects further underscores their importance as a key component in probiotic formulations. Numerous studies have demonstrated the efficacy of bifidobacteria in enhancing gut health, supporting immune function, and preventing gastrointestinal disorders^[45-52]^.

In this review, we will delve into the specific roles and benefits of bifidobacteria as probiotic bacteria, exploring their mechanisms of action, clinical applications, and potential to promote gut health and overall well-being.

## THE PROBIOTICS

The current definition, formulated in 2002 by the expert group of the Food and Agriculture Organization of the United Nations (FAO) and by World Health Organization (WHO), describes probiotics as “live microorganisms that, when administered in sufficient amounts, confer a health benefit on the host”^[53]^. This definition remains widely accepted within the scientific community. The International Scientific Association for Probiotics and Prebiotics (ISAPP) has endorsed this definition, emphasizing its flexibility in encompassing a broad range of products while maintaining key requirements such as viability and sufficient dosage. ISAPP also acknowledges the need for robust scientific evidence for strain-specific benefits, aligning with probiotic research’s evolving nature^[54]^.

Probiotic microorganisms can be consumed naturally through foods, particularly fermented ones, but are also available as supplements or pharmaceutical products. Their integration into our diet can bring a series of benefits to our health. In fact, these beneficial microorganisms exert numerous beneficial effects on the human body, both at systemic and local levels, inhibiting the colonization of pathogenic bacteria^[55]^, reducing intestinal inflammation^[56]^, modulating the host’s immune response^[57,58]^, and promoting intestinal eubiosis^[59,60]^. Additionally, probiotics exhibit a range of other health-promoting effects, including anti-diabetic, antioxidant, anti-aging, antimicrobial, and anti-biofilm properties^[61-63]^. The growing evidence of their health-promoting roles has prompted further investigations into their effects on specific pathologies, including gastrointestinal disorders/diseases, such as IBS, IBD, and antibiotic-associated diarrhea^[64-67]^, suggesting the primary role of probiotic bacteria in the mechanisms involved in restoring the balance of the intestinal microbiota and reducing inflammation^[68-71]^.

Despite the multiple health benefits, research on probiotics has highlighted some limitations, including the poorly understood molecular mechanisms, strain-specific behaviors, short-term effects, niche-specificity (both allochthonous and autochthonous), and the potential transfer of antibiotic resistance genes carried by probiotic bacteria to other members of the human microbiota^[72,73]^. Legislative regulations for the use of probiotics have been introduced to ensure consumer safety. For example, in the United States, microorganisms intended for probiotic products must obtain Generally Recognized As Safe (GRAS) status, regulated by the Food and Drug Administration (FDA). Similarly, in Europe, the European Food Safety Authority (EFSA) has established the concept of Qualified Presumption of Safety (QPS). QPS is a safety assessment approach that adds specific criteria to evaluate bacterial supplements. These criteria include verifying a history of safe use and confirming the absence of traits that could pose safety concerns, such as the potential for transferring antibiotic resistance genes^[74-76]^. However, the current regulation of probiotic bacteria varies significantly from country to country, and no common worldwide regulation exists.

The main probiotic microorganisms known and utilized in supplement products are mainly represented by species belonging to the *Bifidobacterium* and *Lactobacillus* genera, as well as other lactic acid bacteria, such as species belonging to *Lactococcus* and *Streptococcus* genera, but also *Escherichia coli* (*E. coli*) Nissle 1917, *Enterococcus faecium, Bacillus coagulans*, and the yeast *Saccharomyces boulardii*^[77-79]^.

Interestingly, a new frontier of novel-generation probiotics has emerged to obtain the benefits of probiotic organisms without relying on their live forms. The term postbiotics has been broadly defined as preparations containing inanimate microbial cells and/or their metabolites that confer a health benefit on the host^[80]^. This definition includes both inactivated cells and the bioactive molecules they produce, such as enzymes, secreted proteins, short-chain fatty acids (SCFAs), vitamins, biosurfactants, amino acids, and peptides. However, in the very recent scientific literature, a distinction has begun to emerge between postbiotics and parabiotics^[63,81,82]^, with the latter term being used to specifically refer to inactivated microbial cells or their crude extracts, while postbiotics now commonly refers to metabolic products or cell components. Although the term parabiotics is not yet fully recognized across the scientific community, it reflects an evolving understanding of these innovative approaches and their potential health benefits, complementing traditional probiotic use. Nevertheless, probiotics remain the most widely used supplements, particularly due to the strong evidence supporting their beneficial effects.

This review article provides readers with a detailed understanding of bifidobacteria and their potential to improve human health^[83]^. Bifidobacteria, including *Bifidobacterium bifidum* (*B. bifidum*), *Bifidobacterium longum* (*B. longum*), *Bifidobacterium lactis* (*B. lactis*) [formerly known as *Bifidobacterium animalis* (*B. animalis*) subsp. *lactis*], and *Bifidobacterium breve* (*B. breve*), are among the first microbes to colonize the human gastrointestinal tract^[84]^. Their importance is essential from the early days of life. For example, some species present in the intestine from birth can metabolize human milk oligosaccharides (HMOs) present in breast milk, which would otherwise be indigestible by humans^[85-88]^. Overall, the benefits of these probiotic microorganisms are numerous and include modulating the immune response, maintaining the integrity of the intestinal barrier, and competing with pathogenic bacteria for adhesion to host tissue [Figure 1B]. Additionally, bifidobacteria produce various metabolites, such as vitamins, acetate, and lactate^[89-91]^, which can contribute to the production of SCFAs, such as butyrate, through cross-feeding interactions with other gut microbes that metabolize acetate, thereby promoting health benefits^[92,93]^ [Figure 1B]. Furthermore, certain *Bifidobacterium* species have been associated with the production of molecules that act as neurotransmitters, such as gamma-aminobutyric acid (GABA), which may play a role in modulating mental health and stress responses in the host^[94]^ [Figure 1B]. These benefits have triggered significant interest in researching bifidobacteria as potential therapeutic agents in various human health conditions. Therefore, manipulating the intestinal microbiota with bifidobacteria has been proposed as a promising dietary strategy, emphasizing the importance of further research to understand the underlying mechanisms and evaluate the efficacy of such treatments through human clinical studies^[95]^.

## THERAPEUTIC POTENTIAL OF BIFIDOBACTERIAL SPECIES

Based on current scientific literature, which includes well-designed clinical studies and systematic reviews, it is clear that probiotics offer benefits to many different human body sites. The studies discussed in this section were selected through a detailed search of the currently existing scientific literature by using specific keywords such as the names of individual *Bifidobacterium* species, “probiotics”, and specific diseases or conditions. This approach ensured a comprehensive selection of relevant scientific literature on this topic. The team’s domain knowledge and expertise were also crucial in interpreting the findings and finalizing the inclusion of the most scientifically robust studies. The following section will examine studies on various species of the *Bifidobacterium* genus and their roles in specific diseases affecting major human body compartments, such as the gastrointestinal tract, respiratory tract, skeletal and muscular system, and nervous system [Table 1].

**Table 1:** Bifidobacterial species discussed in this review with reported beneficial effects on specific diseases or disorders

	**PMID**	**Study type**	**Population characteristics**	**Bifidobacterial species**
Constipation	35079761	Human	Adults	*B. bifidum*
	33996367	Human	Adults	*B. bifidum*
	33750988	Animal	Murine	*B. bifidum*
	36382178	Human	Adults	*B. longum*
	28884754	Animal	Murine	*B. longum*
Antibiotic-associated diarrhea	36558391	Animal	Murine	*B. bifidum*
	31544979	Animal	Murine	*B. bifidum*
	35031969	Human	Adults	*B. lactis*
	34444974	Human	Adults	*B. lactis*
Acute gastroenteritis-induced diarrhea	26801008	*In vitro*	**-**	*B. adolescentis*
	27375585	*In vitro*	**-**	*B. adolescentis*
	28432676	*In vitro*	**-**	*B. adolescentis*
Stress-induced diarrhea	23200466	Animal	Murine	*B. bifidum*
	25918671	Human	Adults	*B. bifidum*
IBS/IBD	35935215	Animal	Murine	*B. bifidum*
	21418261	Human	Adults	*B. bifidum*
	37240476	Human	Adults	*B. bifidum*
	32277872	Human	Adults	*B. bifidum*
	37702965	Human	Adults	*B. longum*
	21525768	Human	Adults	*B. breve*
*Helicobacter pylori* infection	29573807	Human	Adults	*B. bifidum*
Celiac disease	21651295	*In vitro*	**-**	*B. longum*
	18980693	Human	Children	*B. longum*
	26134988	Human	Children	*B. breve*
	27782071	Human	Children and Adults	*B. breve*
Obesity, hypercholesterolemia, diabetes	24985000	Human	Children	*B. bifidum*
	23872958	Animal	Murine	*B. bifidum*
	25863679	Animal and *in vitro*	Murine	*B. bifidum*
	34365978	Human	Adults	*B. adolescentis*
	35745208	Animal	Murine	*B. adolescentis*
	21914236	Animal	Murine	*B. adolescentis*
	26090097	Human	Adults	*B. breve*
	30094122	Human	Adults	*B. breve*
Respiratory infections and asthma	26372517	Human	Children	*B.lactis*
	36004715	Human	Children	*B.lactis*
	26840903	Human	Adults	*B. adolescentis*
	29633635	Animal	Murine	*B. adolescentis*
Arthritis	31168650	Animal	Murine	*B. bifidum*, *B. longum* and *B. breve*
	32383727	Animal	Murine	*B. adolescentis*
	18848647	Human	Adults	*B. adolescentis*
	17953607	Human	Adults	*B. adolescentis*
	36377740	Animal	Murine	*B.breve*
Anxiety and depression	27801892	Human	Adults	*B.longum*
	32300799	Human	Adults	*B.longum*
	37513541	Human	Adults	*B.longum*
	21988661	Animal and *in vitro*	Murine	*B.longum*
	32485204	Animal	Murine	*B.longum*
	32839473	Animal and *in vitro*	Murine	*B. adolescentis*

*B. bifidum*: *Bifidobacterium bifidum*; *B. longum*: *Bifidobacterium longum*; *B. lactis*: *Bifidobacterium lactis*; *B. adolescentis*: *Bifidobacterium adolescentis*; IBS: irritable bowel syndrome; IBD: inflammatory bowel disease; *B. breve*: *Bifidobacterium breve*.

## GASTROINTESTINAL TRACT

The gastrointestinal tract plays a key role in critical bodily functions, including nutrient absorption, digestion, and immune system regulation. Moreover, it is often affected by a range of diseases and disorders due to a combination of genetic predispositions and environmental factors. These altered conditions include, for example, constipation, antibiotic-associated diarrhea, gastroenteritis, IBS, IBD, *Helicobacter pylori* (*H. pylori*) infections, celiac disease, obesity, and diabetes. Extensive research efforts have demonstrated that *Bifidobacterium*-based probiotics can provide significant benefits in managing and alleviating symptoms associated with these gastrointestinal conditions.

### Constipation

Constipation is a common and significant health concern, commonly described as reduced and/or difficult bowel movements^[96]^.

Numerous studies have emphasized the potential of bifidobacteria as an effective treatment for constipation due to their beneficial impact on regulating intestinal microbiota, improving intestinal motility, and modulating inflammatory responses.

In this context, a human study demonstrated that the intake of *B. bifidum* leads to significant improvements in stool consistency and increased frequency of spontaneous bowel movements in individuals suffering from chronic constipation^[97]^. These effects are associated with the enhancement of the Firmicutes/Bacteroidetes ratio in the intestinal microbiota and increased concentrations of acetic and butyric acid^[97]^. These combined effects seem to lead to a notable improvement in clinical symptoms associated with functional constipation. Furthermore, the consumption of species belonging to the *Bifidobacterium* genus has been shown to significantly alter gut microbial metabolism, particularly influencing carbohydrate metabolism pathways such as propanoate and butanoate through interactions with other gut microbes. While *Bifidobacterium* species themselves do not produce these metabolites, they may facilitate their production by other members of the gut microbiota^[98]^. However, further metagenomic research is needed to identify the metabolic pathways involved precisely^[98]^. Moreover, studies conducted on murine models have further confirmed the effectiveness of *B*. *bifidum* in treating constipation, improving several physiological parameters and, consequentially, intestinal health. Specifically, *B. bifidum* has been reported to promote gut microbiota homeostasis by influencing butyrate production through cross-feeding interactions with other butyrate-producing bacteria. Butyric acid, a metabolite produced by the gut microbiota, has been shown to promote serotonin (5-HT) production by increasing the expression of the serotonin-producing enzyme tryptophan hydroxylase-1 (TPH1) in the host. Additionally, *B. bifidum* has been shown to regulate neurotransmitter levels, such as dopamine and acetylcholine, by suppressing dopamine increases and preventing acetylcholine decreases. These results suggest that *B. bifidum* may contribute to alleviating dysbiosis, enhancing organic acid levels, and improving neurotransmission^[99]^.

Similarly, *B. longum* has been studied for its ability to improve bowel movement frequency, highlighting the importance of a personalized treatment approach based on individual microbial profiles^[100]^. In a human study, the intake of *B. longum* was associated with an increase in bacterial genera belonging to the Clostridia class, which are crucial for producing butyrate and other SCFAs, contributing to increased bowel movement frequency^[100]^. Moreover, murine model studies have further demonstrated the effectiveness of *B. longum* in alleviating constipation through mechanisms such as the modulation of intestinal function and reduction of inflammation^[101,102]^.

### Antibiotic-associated diarrhea

Numerous recent studies have documented the impact of antibiotics on the composition and functionality of the microbiota^[103-105]^. Antibiotics, especially those with a broad spectrum, act on a wide range of microorganisms, including the normal microbiota of an individual, altering intestinal microbiota composition and particularly reducing its diversity. Consequently, antibiotics might disrupt host-microbe interactions, compromising immune system homeostasis and reducing resistance to colonization by pathogenic strains. Intestinal dysbiosis, defined as the disruption of the symbiotic balance between the microbiota and the host that antibiotics may induce, can, in turn, lead to diarrhea and recurrent infections caused by opportunistic pathogens such as *Clostridioides difficile*^[106-109]^.

In murine models, it has been shown that probiotic therapy based on *B. bifidum* alleviates diarrhea symptoms, restores the structure of intestinal villi, and improves microbiota health. Additionally, it has been demonstrated that *B. bifidum* alleviates inflammation and tissue damage in sodium dextran sulfate-induced colitis in murine models^[110]^. Similarly, a specific murine model with diarrhea caused by *E. coli* overgrowth, simulating antibiotic-induced dysbiosis, demonstrated that the supplementation of *B. bifidum* improved dysbiosis and suppressed diarrhea symptoms by reducing the excessive growth of *E. coli* in the intestine^[111]^. These beneficial effects are probably also related to the increased production of IgA induced by *B. bifidum*, which easily binds to *E. coli*, reducing its growth and resolving diarrhea. Furthermore, the analysis of the microbiota composition has shown a trend toward an increase in butyrate-producing bacteria associated with the treatment involving *B. bifidum*. In fact, butyric acid, a SCFA, is used as an energy source by intestinal epithelial cells, contributing to regulating the intestinal epithelium and improving intestinal activity^[111-113]^.

A recent study regarding *B. lactis* has demonstrated its effectiveness in preventing diarrhea and alleviating gastrointestinal symptoms in hospitalized patients undergoing antibiotic treatment, significantly reducing symptoms^[114]^. Moreover, it has been observed that consuming yogurt containing this species during antibiotic treatment mitigates the negative impact of antibiotics on fecal microbiota. This beneficial effect may be attributed to yogurt containing *B. lactis*, which attenuated the decrease in acetate levels following antibiotic treatment and facilitated a quicker return to baseline SCFA levels^[115]^. Furthermore, it has been shown in a murine model that supplementation of *B. lactis* during antibiotic-associated diarrhea led to significant improvement in symptoms, contributing to restoring intestinal microbiota and reducing inflammation^[116]^.

### Acute diarrhea caused by gastroenteritis

Acute gastroenteritis represents one of the major global health issues, particularly in children^[117]^. The primary etiological agents of gastroenteritis in children are *Rotaviruses* and *Noroviruses*^[118-120]^, and the main symptoms include abdominal pain, profuse diarrhea, and vomiting. Although the primary treatment focuses on electrolyte replenishment and rehydration^[121-123]^, the use of selected probiotic microorganisms may also be beneficial in alleviating the symptoms of the infection.

*Bifidobacterium adolescentis* (*B. adolescentis*), for example, exhibits potential antiviral effects against rotavirus and noroviruses. In fact, *B. adolescentis* metabolites could alter the replication of viral particles, specifically by reducing the intracellular amount of NSP4 and Ca^2+^ release, thereby decreasing the virus’s ability to enter cells and replicate^[124-126]^. Furthermore, protein metabolites obtained from *B. adolescentis* cells could prevent the entry of rotavirus by directly affecting the viral particle. The hypothesized mechanism involves the adhesion process to cellular receptors not being efficiently executed due to alterations in the viral outer proteins, such as VP7 or VP4. However, further studies are needed to elucidate this antiviral activity’s mechanisms, including direct interactions with cellular receptors or intracellular regulatory processes^[127]^. This probiotic microorganism has also exhibited antiviral effects against *Coxsackievirus* B3, reducing the number of viral sequence copies. The mechanism of action related to antiviral effects requires further study. Additionally, *B. adolescentis* has been shown to provide protection against intestinal bacterial infections by *Yersinia enterocolitica* in murine models, suggesting that the presence of *B. adolescentis* might offer protection against yersiniosis by enhancing epithelial barrier function through direct interactions with intestinal epithelial cells and by altering the composition of the microbiota^[128,129]^.

In addition to antibiotic-associated diarrhea and diarrhea related to viral infections, several other forms of diarrhea are influenced by other various factors^[130]^.

Among these forms, one of particular interest is stress-induced diarrhea. The brain-gut-enteric microbiota axis may modulate this effect, and it is bidirectional^[131-133]^. Moreover, several studies have demonstrated that *B. bifidum* effectively alleviates gastrointestinal disorders and reduces stress in university students, indicating its positive impact on both psychological and physical well-being^[131]^. Specifically, oral intake of *B. bifidum* has been shown to reduce the incidence of diarrhea. The reduction in self-reported stress through probiotics may result from various mechanisms involving both the immune and endocrine systems, although these mechanisms are yet to be fully elucidated^[131,134-136]^.

### IBD

Numerous studies have examined the effectiveness of *B. bifidum, B. breve,* and *B. longum* in treating symptoms associated with IBD, such as bloating, gas, abdominal pain, cramps, and other digestive disorders^[137-140]^. IBD, which includes both Crohn’s disease (CD) and ulcerative colitis (UC), is of particular interest. IBD should be regarded as a systemic condition, not confined to the gastrointestinal tract alone, as many patients exhibit symptoms outside the intestine. While CD and UC share common pathological and clinical characteristics, each also presents specific distinctive differences^[141]^. These studies provide an overview of the intestinal health benefits these probiotics offer and the mechanisms of action involved.

Regarding *B. bifidum*, several studies have highlighted its role in improving quality of life and alleviating gastrointestinal symptoms in subjects with IBD. A recent murine clinical demonstrated that early treatment with *B. bifidum* can reduce inflammation and promote intestinal mucosal integrity, suggesting a potential protective effect against long-term colitis. The molecular mechanisms involved are not yet fully understood, but the production of specific metabolites, such as acetic and butyric SCFA, can promote the healthy development of the intestine, supporting the growth and health of the intestinal mucosa^[142]^.

As for *B. breve*, several studies have highlighted its efficacy in improving clinical conditions in patients with UC. In detail, *B. breve* intake, either alone or in combination with galacto-oligosaccharides (GOS), led to significant improvements in clinical parameters and a rebalancing of the intestinal microbiota. Moreover, *B. breve* contained in fermented milk with bifidobacteria significantly improved disease conditions in patients with UC, as assessed by colonoscopic index and myeloperoxidase levels. The observed benefits of this strain were at least partly attributed to the modulation of luminal parameters, such as intestinal microbiota and pH^[143]^.

### IBS

Several studies have examined the effectiveness of *Bifidobacterium*-based probiotics in treating symptoms associated with IBS, highlighting their role in improving quality of life and alleviating gastrointestinal symptoms in affected individuals. Specifically, recent studies on *B. bifidum* have demonstrated that a four-week intake significantly reduces the severity index of IBS and improves symptoms such as abdominal pain and dyspepsia. Therefore, *B. bifidum* treatment has significantly alleviated these symptoms, although the underlying mechanisms remain largely unclear^[144-146]^. Moreover, the efficacy of *B. longum* in reducing IBS symptoms has been investigated, focusing primarily on bloating, abdominal pain, and constipation. The results highlighted that an eight-week intake of *B. longum* significantly reduces IBS symptoms^[147]^. An additional clinical trial confirmed that *B. longum* could improve the quality of life and reduce the severity of IBS in affected patients. However, further research still needs to clarify various mechanisms through which B. longum exerts these benefits^[148]^.

### Mitigation of the effects of *H. pylori* infection

*H. pylori* is a Gram-negative, opportunistic pathogen that colonizes the human stomach and is implicated in various gastrointestinal diseases. In fact, *H. pylori* infection can lead to gastric and duodenal ulcers, gastritis, and gastric carcinoma. Eradication of the bacterium through antibiotic therapy causes an alteration of the intestinal microbiota, which can be mitigated with the use of probiotics. Furthermore, probiotics offer additional benefits in this clinical context, extending beyond merely mitigating effects on the microbiota^[149]^.

Supplementation with *B. bifidum* has been shown to alleviate infection symptoms in healthy adults effectively. Significant relief from postprandial discomfort and epigastric pain has been reported after consuming fermented milk encompassing *B. bifidum* cells for four weeks^[150]^. *B. bifidum* has been found to enhance the physical gastric barrier and regulate NF-kB signaling in conditions such as *H. pylori*-associated gastritis, providing new insights into the treatment of gastroesophageal reflux disease and related disorders^[151]^. Additionally, consuming fermented milk containing *B. bifidum* has been reported to improve gastrointestinal symptoms and reduce stress markers in subjects with functional gastrointestinal disorders^[136]^.

Moreover, it has been highlighted that *B. lactis* aids in suppressing *H. pylori*, decreasing bacterial load and gastric mucosal damage in infected gerbils, and lowering inflammatory cytokine levels. These findings suggest that *B. lactis* could be a complementary approach in *H. pylori* infection management^[152]^. Moreover, a significant improvement in symptoms has been observed in women with digestive symptoms such as abdominal pain or discomfort, bloating, flatulence, and stomach rumbling after the intake for two weeks of a fermented milk product containing *B. lactis* ^[153]^.

### Celiac disease

Celiac disease is an autoimmune disease affecting the small intestine. Since gluten is the triggering factor, the only current treatment is a strict gluten-free diet. In recent years, a correlation between intestinal dysbiosis and celiac disease has been observed, leading to the exploration of complementary therapeutic strategies. Among these, modulation of the intestinal microbiota has emerged as a promising area of research^[154]^.

*B. breve* has been investigated for its role in the treatment of celiac disease and food intolerances, and it has been reported that this probiotic bacterium can improve the symptoms of celiac disease by modulating the immune response and altering the fecal microbiota^[155]^. Moreover, several studies demonstrate that *B. breve* strains, in combination with a gluten-free diet (GFD), temporarily reduce TNF-α production in children with celiac disease, thereby counteracting the pro-inflammatory environment. Reducing TNF-α could decrease the intestinal and systemic complications of celiac disease. Further studies are needed to confirm the benefit of this probiotic as a complementary therapy to a GFD^[156,157]^.

Similarly, *B. longum* has shown potential benefits in the treatment of celiac disease. In this context, it has been observed that *B. longum* can reduce gliadin toxicity and modify the response of intestinal cells, suggesting a possible protective role against celiac disease^[158]^. Further studies have confirmed the efficacy of *B. longum* in modulating the immune response, reducing pro-inflammatory cytokines, and improving gluten tolerance in celiac disease models^[159,160]^.

### Obesity, hypercholesterolemia and diabetes

The gastrointestinal tract plays a crucial role in overall metabolic health, influencing conditions such as obesity, hypercholesterolemia, and diabetes. These metabolic disorders are interconnected with gut microbiota composition, where probiotics, particularly those based on *Bifidobacterium* species, have shown promising therapeutic potential.

In this context, it has been demonstrated that administering *B. bifidum* to children with primary dyslipidemia significantly improved total cholesterol and low-density lipoprotein cholesterol (LDL-C) levels^[161]^. Another study investigated 34 human strains of the *Bifidobacterium* genus, which were assessed for their cholesterol adsorption capacity and bile salt hydrolase activity, which represent two strain-specific characteristics. It was found that two strains of the species *B. bifidum* exhibited a significant ability to adsorb cholesterol. The administration of a probiotic formulation containing these strains led to a significant reduction in total cholesterol and LDL-C levels. However, no effects were observed on high-density lipoprotein cholesterol (HDL-C) levels or the HDL-C/LDL-C ratio^[162]^. Similarly, another study revealed the high capacity of *B. bifidum* to assimilate cholesterol, contributing to the reduction of total cholesterol and LDL-C in murine models, and indicated the capability of a specific strain, i.e., *B. bifidum* PRL2010, to reduce cholesterol by converting it into coprostanol, with possible beneficial effects on cardiovascular health^[163]^.

Regarding *B. breve*, significant fat mass reductions and blood parameters improvements related to liver function and inflammation were observed in adults prone to obesity. In detail, several studies suggest that *B. breve* may provide benefits in improving metabolic disorders associated with obesity. *B. breve* can synthesize SCFAs, including acetic acid and lactic acid, and produce other bioactive components, such as conjugated linoleic acid and various fatty acid metabolites. These factors may play a key role in influencing the effects associated with metabolic syndrome^[164,165]^. Additionally, it has been shown that the intake of probiotic microorganisms, including *B. breve*, improves various metabolic risk factors in subjects with metabolic diseases^[166]^.

Furthermore, the beneficial effect of the combination of berberine and *B. adolescentis* has been highlighted in a recent study, which demonstrated a significant reduction in fasting blood glucose levels and glycated hemoglobin, along with an improvement in the composition of the intestinal microbiota in subjects with hyperglycemia^[167]^. Moreover, *Bifidobacterium* strains seemed to alleviate type 2 diabetes symptoms in mice, reducing inflammation and increasing acetic acid and butyric acid levels. These SCFAs enhance energy homeostasis and may mitigate metabolic disorders. Supplementation with *B. adolescentis* or *B. bifidum* significantly increased SCFA levels and reduced blood glucose levels. Furthermore, SCFAs were negatively correlated with glucose, insulin resistance, and inflammatory markers, suggesting that the beneficial effects of these strains are linked to their impact on SCFAs^[168]^. Further research has observed a reduction in visceral fat accumulation and inflammation in murine models, suggesting a potential benefit of *B. adolescentis* in the management of obesity and diabetes^[169,170]^.

## RESPIRATORY TRACT

Historically, the respiratory tract was considered a sterile environment. However, recent studies have revealed a diverse microbial community residing in the respiratory tract, which appears to play a significant role in maintaining human health. Growing scientific evidence suggests a relationship between respiratory microbiota composition and overall respiratory health. Additionally, the gut-lung axis is a concept that describes the bidirectional interaction between the gut and respiratory microbiota. This interaction suggests that probiotics positively affect gut microbiota and may also influence respiratory health by modulating immune responses and microbial communities across these two systems.

### Respiratory tract infections and asthma

Respiratory tract infections and asthma are common conditions that significantly impact global health. Emerging research has shown that probiotics, particularly species from the genus *Bifidobacterium*, may play a beneficial role in managing these conditions.

In this context, a recent study regarding children with respiratory tract infections demonstrated that administering probiotic products containing *B. lactis* twice daily could reduce respiratory infection incidence^[171]^. Moreover, another study explored the effect of controlled administration of *B. lactis* on an infant cohort, observing a decrease in respiratory tract infections during the first eight months of life in those treated with the probiotic compared to the control group^[172]^. Additionally, a randomized, double-blind, placebo-controlled trial assessed the benefits of probiotics in infant formula on upper respiratory tract infections (URTIs) in infants aged six to 15 months. The study examined the effect of the probiotic *B. lactis* on reducing URTIs. The results showed that no infants who received the probiotic developed URTIs or required antibiotic or antiviral treatments, unlike the control group, which experienced significant infection rates, demonstrating a significant reduction in URTIs in the probiotic-treated group^[173]^. Furthermore, *B. adolescentis* has also been associated with a reduction in allergic inflammation and improvements in asthma models. Lower levels of bifidobacteria, including the species *B. adolescentis*, have been identified in adult subjects with allergic asthma, suggesting a potential protective role of bifidobacteria in respiratory diseases^[174]^. Additionally, it has been demonstrated that treatment with *B. adolescentis* reduces allergic inflammation in the airways of murine models, indicating a potential therapeutic effect for allergic asthma^[175]^. However, the mechanisms by which these probiotic microorganisms exert these benefits are not fully understood.

## SKELETAL AND MUSCULAR SYSTEM

The skeletal and muscular systems are fundamental to human movement and structural integrity. They are often affected by several conditions, such as arthritis, significantly impacting quality of life. Recent studies have explored the potential benefits of probiotics, particularly *Bifidobacterium* species, in managing these conditions by modulating the gut microbiota and immune response.

### Arthritis

Arthritis, a chronic inflammatory condition affecting the joints, can severely impact mobility and quality of life. Increasing scientific interest has focused on the potential therapeutic benefits of probiotics, particularly *Bifidobacterium* strains, in managing arthritis. These probiotic bacteria may modulate the immune response and intestinal microbiota, offering new perspectives in the treatment of rheumatoid arthritis (RA) and other forms of arthritis. In particular, the treatment of arthritis through the use of probiotic strains such as *B. adolescentis* and *B. breve* has garnered increasing interest in the scientific community, highlighting potential benefits in animal models and opening new therapeutic perspectives for this chronic inflammatory condition. Notably, the early administration of these probiotics demonstrated better results in promoting the production of SCFAs. Moreover, the early-treated group exhibited a significantly higher frequency of regulatory T cells (Tregs) and lower TNF levels than the group that received *B. adolescentis* at a later stage. Additionally, in the early-treated subjects, SCFAs were positively correlated with Treg levels and negatively correlated with pro-inflammatory cytokines^[176,177]^. Furthermore, recent research has also emphasized that administering *B. adolescentis* can mitigate arthritis development in murine models by modulating the immune response and promoting an increased population of regulatory T cells^[178]^. Moreover, *B. adolescentis* seemed crucial in modulating the immune response and countering specific pathogenic factors associated with RA.

Furthermore, increasing evidence suggests a significant correlation between the development of RA and the periodontal disease, characterized by the presence of *Porphyromonas gingivalis* (*P. gingivalis*). The higher frequency of antibodies against *P. gingivalis* in patients with RA indicates a potential involvement of this bacterium in the disease’s pathogenesis. In this context, the administration of *B. adolescentis* may be beneficial, as it reduces the concentration of vitamin K, a nutrient essential for the survival and virulence of *P. gingivalis*, thereby potentially mitigating its impact on both oral and intestinal microbiota and contributing to the management of RA^[179-182]^.

Similarly, *B. breve* has shown efficacy in repairing the intestinal barrier and reducing systemic inflammation in collagen-induced arthritis murine models^[183]^. This probiotic positively influenced intestinal microbiota composition, increased SCFA levels, and inhibited inflammatory pathways, thereby improving conditions such as arthritis. In a study of arthritic rats, evaluation of serum markers of arthritis, such as C-reactive protein (CRP) and rheumatoid factor (RF), showed that the group treated with *B. breve* had significantly reduced levels of these markers compared to the untreated control rats. Moreover, the administration of *B. breve*, *B. longum*, and *B. bifidum* appeared to significantly reduce serum levels of inflammatory markers and pro-inflammatory cytokines compared to the arthritic control group. Additionally, rats treated with bifidobacteria exhibited lower levels of lipid peroxides, nitric oxides, and protein carbonyls, indicating reduced oxidative stress^[184]^. In conclusion, bifidobacteria were found to reduce the severity and progression of arthritis, with effects varying depending on the strain. Specifically, *B. breve* demonstrated the most pronounced anti-arthritic effects compared to *B. bifidum* and *B. longum*, though all strains provided benefits in treating arthritis in animal models. These studies underscore the importance of further elucidating the underlying mechanisms and assessing the efficacy of such treatments in human clinical trials to validate the potential benefits observed in animal models^[184]^.

## NERVOUS SYSTEM

The nervous system, encompassing the brain and spinal cord, regulates and coordinates body activities. Emerging research has proposed the existence of a gut-brain axis, a bidirectional communication pathway between the gut microbiota and the brain, suggesting that gut health can significantly impact mental health. This concept underpins the potential of probiotics to influence neurological conditions.

### Anxiety and depression

Anxiety and depression are increasingly common mental health disorders that can significantly impair daily functioning and quality of life^[185]^. These conditions are often associated with gastrointestinal issues, highlighting a potential link between gut health and mental well-being^[186,187]^. In this context, the new concept of the gut-brain axis suggests that gut microbiota can influence brain function and mental health, providing a rationale for exploring the therapeutic potential of probiotics in managing anxiety and depression^[186,188]^.

Recent studies have revealed that the gut microbiota can synthesize GABA, i.e., γ-Aminobutyric acid, a key inhibitory neurotransmitter in the regulation of the gut-brain axis^[189,190]^. Alterations in GABA metabolism have been associated with disorders such as anxiety and depression^[190-192]^. A recent genomic analysis conducted on over 1,000 bifidobacteria strains identified *B. adolescentis* as a promising producer of GABA in the human gastrointestinal tract^[94]^. Furthermore, *in silico* analysis of metagenomic data from human and animal studies showed a possible correlation between the presence of *B. adolescentis* and mental disorders, such as depression and anxiety. Moreover, *in vivo* experiments using the murine model further supported these findings. In fact, specific strains of *B. adolescentis*, administered to rats, demonstrated the ability to increase GABA production. These results suggest that *B. adolescentis* could play a significant role in gut-brain axis interactions and contribute to developing new therapeutic strategies for mental disorders by modulating the gut microbiota^[94]^.

Recent research on the efficacy of *B. longum* in the treatment of anxiety and depression has yielded promising results, highlighting the potential of these probiotics to modulate key aspects of mental health. In detail, *B. longum* has been demonstrated to possess the ability to mitigate stress response and promote cognitive improvements, reducing cortisol production and enhancing visuospatial memory^[193]^. Similarly, it has been shown that *B. longum* improved mental flexibility and reduced stress in the elderly, suggesting a positive impact on their mental well-being^[194]^. Furthermore, another study described that supplementation with *B. longum* significantly reduces perceived stress and improves sleep quality, highlighting the role of this probiotic bacteria in enhancing stress management and overall mental health^[195]^. Additional research efforts have been made to explore the physiological and behavioral effects of *B. longum*, revealing that this probiotic microorganism modifies neural oscillations in response to social stress, suggesting an impact on brain functions associated with stress management^[196]^. Moreover, further studies have elucidated the mechanism through which *B. longum* influences anxious behavior and the functioning of the hypothalamic-pituitary-adrenal axis, providing a basis for understanding how probiotics can directly influence stress-related behavior and physiology^[197,198]^.

## CONCLUSION

Bifidobacteria, as probiotics, offer substantial therapeutic benefits across various health conditions by modulating the intestinal microbiota and immune responses^[58,199]^. A significant proportion of clinical studies on probiotics focus on bifidobacteria, highlighting their essential role in human health^[200,201]^.

Bifidobacteria have co-evolved with humans and are among the first microorganisms colonizing the infant’s gut and playing a crucial role in the early development of the intestinal microbiota^[11]^. They are essential for educating the immune system and supporting gut physiology, such as stimulating mucin production^[92,202]^. Additionally, bifidobacteria are maintained in the human gut due to the production of prebiotic molecules like mucin^[36,38]^, which support their survival. Moreover, recent studies have reported the transmission of these bacteria from mother to child through vertical transmission mechanisms^[40]^, again underscoring their co-evolution with the human host. The loss of bifidobacteria in both children and adults has been negatively correlated with numerous human diseases and disorders^[51,138,200]^, further emphasizing their critical role in maintaining gut health. In this context, the beneficial effects of bifidobacteria in the gastrointestinal tract are well documented.

In summary, bifidobacteria are fundamental to human health, having co-evolved with their host and playing a critical role from early life. Their ability to modulate the gut microbiota, support immune function, and maintain intestinal barrier integrity makes them indispensable in promoting health and preventing disease. This review underscores the significant therapeutic potential of *Bifidobacterium* species across various body compartments and highlights the necessity for further research to understand the underlying mechanisms and validate clinical efficacy. Additionally, more studies are necessary to identify the genetic bases underlying these beneficial effects and to better understand the interactions with the host.

## DECLARATIONS

### Copyright

© The Author(s) 2024.
